# Intraspecific variation of scent and its impact on pollinators’ preferences

**DOI:** 10.1093/aobpla/plad049

**Published:** 2023-07-21

**Authors:** Mayumi Vega-Polanco, Lislie Solís-Montero, Julio C Rojas, Leopoldo Cruz-López, David Alavez-Rosas, Mario Vallejo-Marín

**Affiliations:** El Colegio de la Frontera Sur (ECOSUR), Unidad Tapachula, Carretera Antiguo Aeropuerto km 2.5, AP 36, Tapachula C.P. 30700, Chiapas, México; El Colegio de la Frontera Sur (ECOSUR), Unidad Tapachula, Carretera Antiguo Aeropuerto km 2.5, AP 36, Tapachula C.P. 30700, Chiapas, México; Consejo Nacional de Humanidades, Ciencias y Tecnologías (CONAHCYT), Av. Insurgentes Sur 1582, Colonia Crédito Constructor, Delegación Benito Juárez, Ciudad de México C.P. 03940, México; El Colegio de la Frontera Sur (ECOSUR), Unidad Tapachula, Carretera Antiguo Aeropuerto km 2.5, AP 36, Tapachula C.P. 30700, Chiapas, México; El Colegio de la Frontera Sur (ECOSUR), Unidad Tapachula, Carretera Antiguo Aeropuerto km 2.5, AP 36, Tapachula C.P. 30700, Chiapas, México; Laboratorio de Ecología de la Conducta de Artrópodos, Instituto de Ecología, Universidad Nacional Autónoma de México, Circuito exterior s/n anexo al Jardín Botánico, Ciudad Universitaria, Ciudad de México C.P. 04510, México; Division of Plant Ecology and Evolution, Department of Ecology and Genetics, Evolutionary Biology Centre, Uppsala University, Norbyvägen 18 D, 752 36 Uppsala, Sweden

**Keywords:** *Bombus impatiens*, chemical composition, pollinators, population variation, volatile compound

## Abstract

Floral scents shape plant–pollinator interactions. Although populations of the same species can vary in their floral scent, little is known about how this variation affects pollinator visitation. In this study, we compare the scents emitted by buzz-pollinated *Solanum rostratum* (Solanaceae) in two areas of its distribution (Mexico and USA) and investigate how these differences in scent affect pollinator preferences and attraction. We determined the variation of floral volatile compounds using hexane extraction followed by gas chromatography coupled with spectrometry. We also performed a field cage multiple-choice bioassay and a Y-tube behavioural bioassay using *Bombus impatiens*. We recorded 13 volatile compounds in floral extracts for plants from both ranges that varied qualitative and semi-quantitatively among populations. We found that in the field cage experiment, bumble bees visited plants from the US populations more frequently than plants from Mexican populations. However, bees showed no difference in preference between extracts from Mexican or US flowers. We conclude that although bees show differential visitation to whole plants of different regions, variation in floral extract alone does not translate into differences in preference by *B. impatiens*. The potential effects of variation in floral scent on the other native bee pollinators remain to be assessed.

## Teaser

Floral scents are key in plant-pollinator interactions. These scents can vary even among populations of the same species, which influences pollinator attraction. In this study, we determined the variation in floral scents in a buzz-pollinated *Solanum rostratum* in two areas of its distribution (Mexico and the USA). Moreover, we investigated how these differences affect pollinator preferences and attraction of Bombus impatiens. Our main finding is that floral scents emitted by *S. rostratum* plants from the Mexican range differ from the USA distribution range; although we found similar preferences of bumble bees for floral extracts, bumble bees preferred to visit plants from the US populations in a field cage bioassay. These contradictory results can probably be attributed to other signals not evaluated in this study or the extraction method of floral extracts. Future studies are necessary to evaluate the potential effects of variation in floral scent on other native bee pollinators.

## Introduction

Floral scents play a key role in plant–pollinator interactions and plant reproductive success (Parachnowitsch *et al.* 2012; Burkle *et al.* 2020). These scents are emitted by different floral structures (i.e. petals, glandular regions or androecium; Dobson 1994; Raguso and Gottesberger 2017). Plants rely on floral scents to ensure their reproduction by increasing emissions when pollinators are more active (Dötterl *et al.* 2005; Friberg *et al.* 2014; Jürgens *et al.* 2014). Floral scents can signal landing and rewarding sites for pollinators, and attract from a long distance (Knudsen *et al.* 2006; Raguso 2008*a*; Raguso and Gottesberger 2017).

Chemical identity, ratios or concentrations of floral scents compounds may vary within plant species, populations or individuals (Raguso *et al.* 2003; Hoballah *et al.* 2005; Knudsen *et al.* 2006; Theis *et al.* 2007; Dötterl *et al.* 2012; Parachnowitsch *et al.* 2012; Delle-Vedove *et al.* 2017). Intraspecific variation has been assessed at two scales, namely the geographical scale and the temporal scale (Dötterl *et al.* 2005; Soler *et al.* 2011; Parachnowitsch *et al.* 2012; Delle-Vedove *et al.* 2017).

On the geographical scale, the literature reports two main types of variation: within individuals of a given population or among close or far populations (e.g. a few kilometres away or located on different continents; Dötterl *et al.* 2005; Soler *et al.* 2011; Parachnowitsch *et al.* 2012). For example, some studies have evaluated geographical variation of floral scents by comparing two or more populations at the same location (Parachnowitsch *et al.* 2012), within the same country (de Manincor *et al.* 2022) or on different continents (Dötterl *et al.* 2005; Soler *et al.* 2011). On temporal scale, other studies have investigated the floral scent variation throughout the day and the night (Balao *et al.* 2011; Dötterl *et al.* 2012), over a diel period (Majetic *et al.* 2015; Powers *et al.* 2022), during flower ontogeny (Martin *et al.* 2017; Romano *et al.* 2022), postpollination (Schiestl *et al.* 1997; Schiestl and Ayasse 2000; Theis and Raguso 2005; Muhlemann *et al.* 2006) and over the years (Burkle *et al.* 2020).

Despite the importance of floral scent as essential signals for pollinator attraction, attention has primarily been given to flower colour or morphology (Fenster *et al.* 2004; Dicke 2006; Raguso 2008*a*, *b*; Whitehead and Peakall 2009). Only a limited number of studies have reported the intraspecific floral scent variation in some specialist plant species (Mant *et al.* 2005; Vereecken and Schiestl 2008, 2009; Hossaert-McKey *et al.* 2010; Ibáñez *et al.* 2010; Soler *et al.* 2011; Breitkopf *et al.* 2013; Suinyuy *et al.* 2015; Delle-Vedove *et al.* 2017; Souto-Vilarós *et al.* 2018; Friberg *et al.* 2019; Petrén *et al.* 2021), and generalist pollinated species (Shuttleworth and Johnson 2010; Suinyuy *et al.* 2015; Larue-Kontic and Junker 2016; Friberg *et al.* 2019; Petrén *et al.* 2021; de Manincor *et al.* 2022). The extent, of floral scents adaptation to local pollinators has been studied more in specialist plants than in generalist plants, likely because it is easy to document pollinators’ community and their response to scents (de Manincor *et al.* 2022).

Plants presenting relatively specialized pollination systems are excellent models for studies aiming to explain the intraspecific floral scent variation because of their close relation with their pollinators. *Solanum rostratum* Dunal (Solanaceae) is an appropriate model to assess the intraspecific variation on a geographical scale because this plant species is considered cosmopolitan. *Solanum rostratum* is a self-compatible annual weed with a complex floral morphology (heteranthery and enantiostyly) that depends on middle and large buzzing bees to reproduce (Bowers 1975; Vallejo-Marín *et al.* 2013; Solís-Montero *et al.* 2015). It is native to Mexico, but it has spread as an invasive species to the USA, Canada, and many other countries in Eurasia, Africa, and Oceania (Whalen 1979; Zhong *et al.* 2009; Zhao *et al.* 2013; Wang *et al.* 2020; Solís-Montero *et al*. 2022). It is known that invasive populations are less genetically diverse than native populations (Zhao *et al.* 2013). However, invasive populations of *S. rostratum* exhibit high outcrossing rates (Solís-Montero 2013; Zhang *et al.* 2017; Zhang and Lou 2018) similar to those of native populations (Vallejo-Marín *et al.* 2013). In native populations of *S*. *rostratum*, effective (i.e. they contact stigma and transfer pollen) and efficient (i.e. they produce fruits and seeds) pollinators are buzz-pollinating bees, mainly of genera *Bombus*, *Xylocopa* and *Centris* (Solís-Montero *et al.* 2015; Vega-Polanco *et al.* 2020); whereas in invasive populations, some species of these genera also act as effective and efficient pollinators (Harris and Kuchs 1902; Linsley and Cazier 1963; Bowers 1975; Jesson and Barrett 2005; Qiu *et al*. 2013; Zhang and Lou 2015). This suggests that recruiting buzz-pollinators in invaded *S. rostratum* regions could favour outcrossing (Solís-Montero *et al.* 2022). These new interactions could be established through modification of its functional floral traits, such as floral morphology or scents, as reported in previous studies with other plant species (Junker *et al.* 2011; Soler *et al.* 2011; Kaiser-Bunbury *et al.* 2014; Larue *et al.* 2016).

In this study, we hypothesized that scents emitted by flowers of a specialist plant species differed between two distribution ranges affecting pollinators’ preference and attraction due its close relationship. The study aimed to answer the following questions: (a) Do the floral scent profile of *S. rostratum* populations vary in the two geographic distribution ranges? (b) How do the floral scents of the two distribution ranges of *S. rostratum* influence the pollinators’ preference?

## Material and Methods

### Studied species

#### Plant material.

*Solanum rostratum*, (also known as buffalobur) grows in dry and disturbed habitats (Whalen 1979; Nee 1993; Vallejo-Marín *et al.* 2013). This plant species has nectarless bright yellow flowers and offers pollen as the only reward to pollinators (Bowers 1975). Its heterantherous flowers have two morphologically and functionally distinct sets of anthers (Whalen 1979; Vallejo-Marin *et al.* 2014). Four yellow anthers, the feeding anthers (FA), are located in the centre of the flower and provide pollen to visiting insects (Bowers 1975; Vallejo-Marín *et al.* 2009). The pollinating anther, a single dark anther, deflected either to the right or to the left of the floral axis, contributes disproportionately to ovule fertilization, and it is usually ignored by floral visitors (Bowers 1975; Vallejo-Marín *et al.* 2009, 2014). In this species, anthers are the structures responsible for the floral scent emission (Solís-Montero *et al.* 2018). *Solanum rostratum* flowers are enantiostylous with style opposite to the pollinating anther; thus, it has two floral morphs (left- and right-deflected style and pollinating anthers (PA); Todd 1882; Jesson and Barrett 2005).

In this study, we used four populations distributed in North America: two populations from Mexico (native range) and two populations from USA (invasive range), widely separated from each other (i.e. about 2581 km). The Mexican populations were in Puebla State: Libres (19°23ʹ25.8″N, 97°14ʹ13.8″W, 2373 m asl; hereafter MX 1) and Amalucan (19°02ʹ52.6″N, 98°07ʹ59.4″W, 2234 m asl; hereafter MX 2). Seeds of these populations were collected in July 2011 and July 2018, respectively. These populations were separated by a distance of 103 km.

The US populations were in two localities in Kansas: 38°58ʹ26.4″N, 96°12ʹ35.9″W, 427 m asl hereafter USA 1 and 38°53ʹ60″N, 96°12ʹ35.9″W, 405 m asl hereafter USA 2. These populations were collected in July 2011; they were separated from each other by 12 km.

In the Mexican populations, MX 1 population is visited mainly by an illegitimate non-buzzing visitor (*Apis mellifera;*Solís-Montero *et al.* 2015), whereas MX 2 is mainly visited by a legitimate buzzing visitor (*Bombus sonorus*; Vega-Polanco *et al.* 2020). Although there is no record of pollinator observation in the US populations, these populations present high outcrossing rates probably associated with local pollinator recruitment (Solís-Montero 2013; Solís-Montero *et al.* 2022). *Bombus impatiens,* a native bumble bee of the USA, is an effective pollinator of *S. rostratum* because this bumble bee contacts the stigma when visiting its flowers (Bowers 1975; Jesson and Barrett 2005).

We collected seeds from 15 randomly chosen individuals per population (4 populations) and sampled 5 fruits per plant. Seeds collected from the individual represent a maternal family (i.e. 15 seeds × 6 maternal families × 4 populations = 360 seeds). These seeds were stored in waxed paper bags at 4 °C and promoted germination by placing them in 1 mL of a 1000 ppm gibberellic acid solution in a 1.5 mL Eppendorf microtube, leaving them for 24 h at 25 °C (Vallejo-Marin *et al.* 2013). The seeds were sown in germination trays with *Sphagnum* Peat Moss substrate (Premier Tech Horticulture, Canada) in a greenhouse at El Colegio de la Frontera Sur (ECOSUR), Tapachula Campus, Chiapas, Mexico (14°53.175ʹN 92°17.195ʹW, altitude 135 m asl). The germination trays were kept under greenhouse conditions at a mean temperature of 34 °C; mean relative humidity of 55 % and a photoperiod of 12 h L:12 h D. After 3 weeks, we transplanted seedlings into 10 cm diameter pots with the same substrate. The final transplants were made in 15 cm diameter pots using the same substrate with a slow-release fertilizer (17-17-17 Excelso, Vigoro, USA). We sampled fresh undamaged flowers on the third day of opening, between 1000 and 1200 h, to make floral extracts for characterizing *S. rostratum* volatiles and pollinator preference bioassays.

#### Bumble bees.

*Bombus impatiens* is a generalist pollinator that forages on hundreds of plant species and numerous plant families (Leonhardt and Blüthgen 2012). *Bombus impatiens* is one of the primary pollinators of *S. rostratum* in its non-native range of distribution (Bowers 1975; Jesson and Barrett 2005). This bumble bee has been used in Mexico since 1994 as an efficient pollinator of tomato crops (Winter *et al.* 2006; Torres-Ruiz and Jones 2012), although it also pollinates several other crops (Artz *et al*. 2011; McGrady 2018). The colonies of *B. impatiens* used in this study were obtained from a commercial supplier (Koppert Mexico, El Marqués, Querétaro). *Bombus impatiens* colonies are maintained under artificial rearing conditions (Díaz 2011, Torres-Ruiz *et al.* 2011).

The queenless colony used in this study contained about 80 to 100 adult workers and an unknown number of developing larvae. The bumble bees were kept under laboratory conditions at 25 ± 2 °C, 80 ± 10 % RH and a photoperiod: 12 h L:12 h D, inside a cage of metal rods lined with tulle (65 cm high × 43 cm wide). Bumble bees were fed with honey and commercial multifloral pollen. Pollen comes from beehives that feed on various flowers in the mountainous region of Motozintla, Chiapas (Baza 2018).

### Characterization of volatiles in floral structures of *S. rostratum*

We collected samples corresponding to populations of *S. rostratum* from the Mexican and USA distribution ranges, grown in a greenhouse at ECOSUR. Each sample corresponded to ten undamaged, fresh and with the same age (3 days after anthesis) flowers or anthers collected between 1000 and 1200 h from eight individuals per population (80 flowers, 320 FA or 80 PA). This sample was placed in vials with 10 mL of hexane (HPLC, Aldrich Toluca, Mexico). Next, we macerated the plant material (flowers or anthers) in the vials with a glass stirrer, and we waited for it to precipitate for 5 min. Then, we decanted the floral extract into a clean vial. We obtained three types of extracts: (a) whole flower (FC), (b) FA and (c) PA (Solís-Montero *et al.* 2018). We obtained two replicates of each type of extract per range (12 samples). Each flower extract was concentrated to 50 µL through a gentle stream of N_2_ air at 25 ± 1 °C, 1 atm and 80 ± 5 % RH. From each type of floral extract, we collected two replicates. The extracts were kept in a sample box at 6.5 °C until use. We collected volatiles from the different types of extracts using hexane extraction.

Floral structure extracts were analysed using a Shimadzu model GC-2010 Plus gas chromatograph (Shimadzu Corp., Japan), coupled to a Shimadzu GCMS-TQ8040 mass spectrometer (Shimadzu Corp., Japan), equipped with a non-polar capillary column (DB5-MS: 30 m × 0.25 mm and 0.25 μm coat thickness, Agilent Technologies, USA; containing 95 % dimethylpolysiloxane and 5 % diphenyl siloxane). The temperature program was 50 °C (2 min) followed by 15 °C min^−1^ increasing to 280 °C (10 min). We used helium as carrier gas, with a constant flow of 1.0 mL min^−1^ with the injector temperature of 250 °C. Ionization was performed by electron impact at 70 eV and 250 °C. We identified preliminary compounds by verifying retention index and mass spectra from the NIST mass spectra library version 2.5 (National Institute of Standards and Technology, Varian, USA) and the Saturn program (Varian). The compound identity was confirmed by comparing mass spectra and retention times with the synthetic standards available from Sigma-Aldrich (Toluca, Mexico), which are >97 % pure, according to the supplier. We calculated the relative abundance of a compound as the ratio of its area to the total area of all the compounds in the sample for each type of extract. Later, we calculated the mean relative abundance for each compound in each floral structure extract.

## Behavioural Bioassays

### *Bombus impatiens* preference for *S. rostratum* in a field cage

The preference bioassays were performed at 31.8 ± 0.31 °C, 52 ± 1 % HR and homogeneous lighting (0.3 lux of intensity) to favour bumble bee discrimination performance. First, we trained the bumble bees for 2 days in a cage (73.5 cm long × 43 cm wide × 65 cm high) of metal rods lined with mesh using one plant from Mexico and other from USA. Then, we conducted a multiple-choice bioassay to evaluate the preference of *B. impatiens* for plants from *S. rostratum* populations in cylindrical field cage (2 m high by 3 m in diameter) made of crystal-coloured anti-aphid mesh **[**see Supporting Information—**Fig. S1**]. In each experimental trial, we randomly placed four flowering plants (two plants from the Mexican populations and two plants from the US populations) at the same height within the field cage. We introduced four bumble bees chosen randomly. We separated the plants at 40 cm from each other and set them in a semicircle [**see Supporting Information—Fig. S1A**]. On each plant, we marked three undamaged flowers of both morphs with paper labels (left and right style; **see Supporting Information—Fig. S1B**); the remaining flowers were bagged with small mesh bags.

Pollinator observations were conducted only on tagged flowers between 0800 and 1000 h, the peak foraging performance period (Bowers 1975). Each observation lasted over a 20-min period, with a 5-min shift to the next observation. We recorded the following parameters: (1) the number of visits; (2) the duration of each visit and (3) whether the flower visitor contacted the sexual organs [**see Supporting Information—Fig. S1C**]. Bumble bees were marked on the thorax with water-based paint after each bioassay in order to avoid using it again. In total, we performed ten trials.

### *Bombus impatiens* preference for *S. rostratum* floral extracts in Y-tube olfactometer

We evaluated the preference of *B. impatiens* for the floral extracts from *S. rostratum* populations in the two distribution ranges in double-choice bioassays in a transparent acrylic olfactometer type ‘Y’ (Solís-Montero *et al.* 2018). The olfactometer has 60 cm of a long base, each arm 30 cm long, square in section with a width of 11 cm. In each arm, a filter paper strip (3 cm × 0.5 cm) was placed with 2 µL of extract of floral structures (0.8 flower equivalent) or with hexane (control). A stream of clean air (1 L min^−1^) was allowed to flow over the paper strips (Solís-Montero *et al.* 2018).

The olfactometer was placed in a square wooden cage, covered with a black cloth and red light (**see Supporting Information—Fig. S2**; Schaeffer *et al*. 2019). The lighting in a wood box was 0.7 lux of intensity. Naive bumble bees fasted for 24 h were released individually on the long arm of the olfactometer and observed for 5 min. We considered a choice when the bumble bee crossed the intersection of the olfactometer to choose one arm. We recorded each arm’s initial choice and residence time over 5 min for each bumble bee. Each trial reversed the assigned treatments for each arm of the olfactometer. At the end of each trial, we cleaned the olfactometer with 70 % ethanol and let the clean air stream flow at 1 L min^−1^ for 1 min, and we changed the position of the two extracts.

We performed comparisons between the floral structures extracts and control (hexane), and between extracts (see Fig. 3). The bioassays were performed at 26 ± 2 °C, 58 ± 5 % RH between 0800 and 1000 h, corresponding to the foraging period of the bumble bee (Bowers 1975). At the end of each trial, we marked the abdomen of each bumble bee with water-soluble paint (Vinci 25 mL, Estado de Mexico, Mexico) to visually identify the individuals in the experiments (Baird *et al.* 2010; de Jager *et al.* 2017) and avoid bias. Water-soluble paint does not affect bumble bees’ behaviour (Switzer and Combes 2016). We carried out 15 to 30 repetitions of each comparison.

## Statistical Analysis

We performed two separated GLM models to analyse the difference in the number of bee bumble visits and visit duration on *S. rostratum* populations in the field cage assays. The first model was fit with Poisson error, and the second one was fit with Gaussian error. In both models, the explanatory variable was range. Means were separated by Tukey contrast using ghlt function from multcomp library (Hothorn *et al.* 2008). Y-tube bioassays data were analyzed using a G-test. The null hypothesis was that floral extracts have no effect on bumble bee's preference. We excluded non-responding bumble bees from data. The contribution of volatiles of the floral extracts in each population was analysed using two classification models, random forest and canonical correlation using randomForest (Genuer *et al.* 2010) and candisc (Friendly and Fox 2016) packages, respectively. All analyses were performed using R software version 4.1.0 (R Core Development Team 2019).

## Results

### Volatile compounds in floral structures of *S. rostratum*

We recorded 13 volatiles, including one unidentified compound, in extracts of floral structures belonging to *S. rostratum* (Table 1). The 12 volatiles identified belong to seven different classes: three alkanes, two alcohols, one ester, one sesquiterpene, one phenylpropene, one lactone, one monoterpene ketone and two sesquiterpenoids. Seven volatiles were presented in all four (Table 1). Eugenol, methyl eugenol and γ-decalactone were only present in floral structures from Mexican populations (Table 1). In contrast, *trans*-geranylacetone was only present in US populations.

**Table 1.** Relative abundance (mean proportion ± standard error) of VOCs found in floral extracts of *Solanum rostratum*, belonging to populations of two geographic distribution ranges of the species. The floral extracts correspond to the whole flower, feeding anthers and pollinating anthers. Mexican populations: MX 1 (Libres) and MX 2 (Amalucan). US populations: USA 1 (Kansas I) and USA 2 (Kansas II) (*n* = 10 replicates for each population). RT = retention time, RI = retention index of compounds. Superindex^*^: VOCs previously reported by Solís-Montero *et al.* 2018; ^a^VOCs reported by Vega-Polanco *et al.* 2020 (collected with SPME).

ID	Compounds	RT (min)	RI	Feeding anthers				Pollinating anthers			
				MX 1	MX 2	USA 1	USA 2	MX 1	MX 2	USA 1	USA 2
1	Dodecane	8.560	1201	12.31 ± 1.93	24.20 ± 4.59	15.19 ± 0.35	20.50 ± 1.79	10.97 ± 4.91	13.88 ± 2.16	32.69 ± 0.29	34.09 ± 3.77
2	Methyl salicylate^*^	8.610	1206	3.06 ± 0.64	2.18 ± 0.16	3.59 ± 0.15	4.41 ± 0.64	3.62 ± 1.40	5.13 ± 0.57	6.98 ± 0.10	6.30 ± 1.33
3	Tetradecane	9.555	1301	3.48 ± 0.87	2.68 ± 0.05	6.28 ± 0.56	8.42 ± 0.31	4.05 ± 1.11	4.03 ± 0.59	13.32 ± 1.27	14.01 ± 0.26
4	Eugenol^*,a^	10.150	1365	2.54 ± 0.75	1.34 ± 0.06			6.80 ± 3.00	6.17 ± 0.59		
5	Copaene^*,a^	10.425	1495	1.20 ± 0.00	0.52 ± 0.03	0.58 ± 0.03	0.46 ± 0.08	2.12 ± 0.94	1.11 ± 0.19	1.62 ± 0.56	1.36 ± 0.22
6	Methyleugenol^*,a^	10.530	1406	0.58 ± 0.04	0.30 ± 0.12			1.19 ± 0.48	1.94 ± 0.74		
7	{152 [M]+, 151 (100); 123 (35); 109 (35); 81 (30)}	10.590	1413	7.61 ± 0.82	3.65 ± 0.35	0.26 ± 0.03	0.32 ± 0.06	5.27 ± 1.01	2.73 ± 0.74	0.75 ± 0.06	1.28 ± 0.03
8	*trans*-Geranylacetone	10.495	1454			27.78 ± 16.29	21.79 ± 3.38			16.36 ± 8.12	16.23 ± 2.53
9	γ-Decalactone^*,a^	11.170	1479	17.61 ± 7.60	24.17 ± 1.61			11.10 ± 5.73	21.03 ± 10.57		
10	Hexadecane^*,a^	12.170	1600	1.92 ± 0.04	1.70 ± 0.41	4.98 ± 0.39	5.93 ± 0.19	4.37 ± 3.13	2.31 ± 0.27	7.35 ± 0.37	8.19 ± 0.22
11	(*E,E*)-Farnesol^*^	13.135	1726	42.02 ± 0.85	44.85 ± 0.16	13.12 ± 3.29	14.00 ± 0.49	9.12 ± 5.76	34.69 ± 1.31		
12	(*E,Z*)-Farnesol	13.315	1750	0.95 ± 0.33	1.20 ± 0.47	28.23 ± 12.49	24.96 ± 0.90	1.84 ± 0.51	2.67 ± 0.25		4.69 ± 1.94
13	Pentadecanol	13.355	1756	6.70 ± 2.58	8.88 ± 1.60			27.64 ± 11.66	0.97 ± 0.16	12.86 ± 0.85	13.84 ± 1.34

ID	Compounds	RT (mín)	RI	Whole flower							
				MX 1	MX 2	USA 1	USA 2				
1	Dodecane	8.560	1201	5.82 ± 2.54	6.18 ± 0.32	22.13 ± 1.91	42.43 ± 29.82				
2	Methyl salicylate^*^	8.610	1206	6.92 ± 1.10	8.53 ± 0.49	14.58 ± 2.90	5.29 ± 0.01				
3	Tetradecane	9.555	1301	6.38 ± 3.87	4.54 ± 2.57	7.44 ± 3.23	2.84 ± 0.00				
4	Eugenol^*,a^	10.150	1365	2.97 ± 0.15	8.29 ± 0.16						
5	Copaene^*,a^	10.425	1495	0.36 ± 0.00	1.31 ± 0.68	0.67 ± 0.02	9.37 ± 0.00				
6	Methyleugenol^*,a^	10.530	1406	0.94 ± 0.00	1.99 ± 0.42						
7	{152 [M]+, 151 (100); 123 (35); 109 (35); 81 (30)}	10.590	1413	1.78 ± 0.49	2.96 ± 0.68	1.65 ± 0.63	0.97 ± 0.00				
8	*trans*-Geranylacetone	10.495	1454			21.92 ± 8.31	14.62 ± 8.80				
9	γ-Decalactone^*,a^	11.170	1479	28.39 ± 3.99	18.91 ± 3.32						
10	Hexadecane^*,a^	12.170	1600	2.05 ± 0.53	2.34 ± 1.42	2.75 ± 0.06	3.61 ± 0.89				
11	(*E,E*)-Farnesol^*^	13.135	1726	33.18 ± 1.16	35.81 ± 0.44	0.64 ± 0.04	3.64 ± 0.01				
12	(*E,Z*)-Farnesol	13.315	1750	1.07 ± 0.06	2.14 ± 0.20	28.22 ± 8.82	17.23 ± 1.98				
13	Pentadecanol	13.355	1756	10.13 ± 3.95	7.00 ± 4.34						

The volatile composition of floral extracts from FA, PA and whole flower (FC) differed among Mexican populations from the US populations. Pentadecanol was only present in the PA of USA 1 and USA 2, while (*E,E*)-farnesol was absent in these anthers. (*E,E*)-Farnesol, γ-decalactone and dodecane were present in highest relative amounts in all floral structures in the Mexican populations (Table 1). Dodecane, (*E,Z*)-farnesol and *trans*-geranylacetone were present in higher relative amounts in all floral structures of the US populations (Table 1).

**Figure 1.**
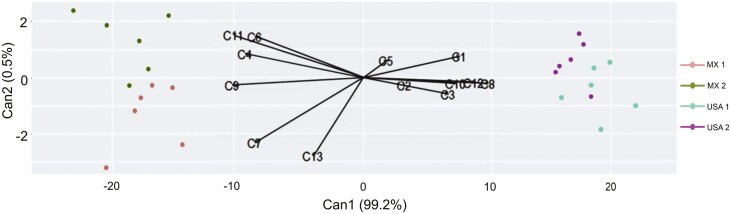
Random forest with a canonical correlation of relative proportion of volatile compounds in floral extracts from Mexican and US populations. C1: dodecane, C2: methyl salicylate, C3: tetradecane, C4: eugenol, C5: α-copaene, C6: methyleugenol, C7: {152 [M]+, 151 (100); 123 (35); 109 (35); 81 (30)}, C8: *trans*-geranylacetone, C9: γ-decalactone, C10: hexadecane, C11: (*E,E*)-farnesol, C12: (*E,Z*)-farnesol and C13: pentadecanol.

The random forest with canonical correlation analysis of extracts revealed 99.7 % of the variance. The first canonical function explained 99.2 % of the variance in the chemical composition, while the second one explained 0.5 % of this variance. Both canonical functions showed that the chemical composition separates the US populations from Mexican populations (Fig. 1; **see Supporting Information—Table S1**). (*E,E*)-Farnesol and γ-decalactone better defined Mexican populations, while (*E,Z*)-farnesol and *trans*-geranylacetone defined US populations (Fig. 1; **see Supporting Information—Table S1**). In this study, we found intraspecific variation of volatile profile in flowers of *S. rostratum* plants in both Mexican and US populations.

## Behavioural Bioassays

### *Bombus impatiens* preference for *S. rostratum* in a field cage

**Figure 2.**
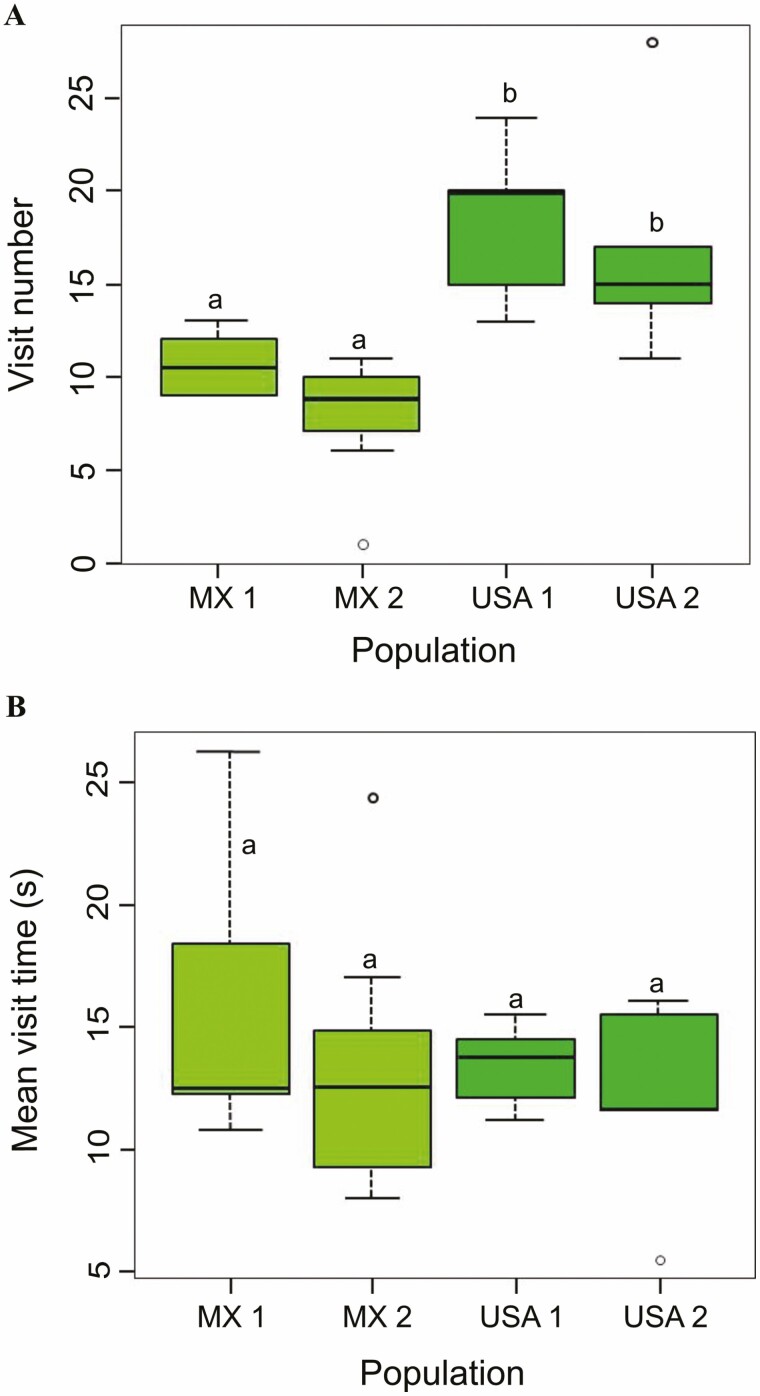
Preference of *Bombus impatiens* for plants of *Solanum rostratum* from populations of two distribution ranges in a multiple-choice behavioural bioassay. (A) The number of visits and (B) the average visit duration of *S. rostratum*. Populations from the Mexican distribution range, MX 1: Libres and MX 2: Amalucan; and US populations, USA 1: Kansas I and USA 2: Kansas II. Different letters indicate statistical differences between treatments (*n* = 196 bumble bees).

Bumble bees visited plants from US populations more than those from Mexican populations (deviance = 34.79, DF = 3, *P* ˂ 0.001; Fig. 2A). There is no difference in the time visiting flowers of plants from the four populations (deviance = 33.60, DF = 3, *P* = 0.71; Fig. 2B). All bumble bees vibrated and contacted reproductive structures in the experiment.

### *Bombus impatiens*: preference for floral extracts of *S. rostratum* in Y-tube olfactometer

**Figure 3.**
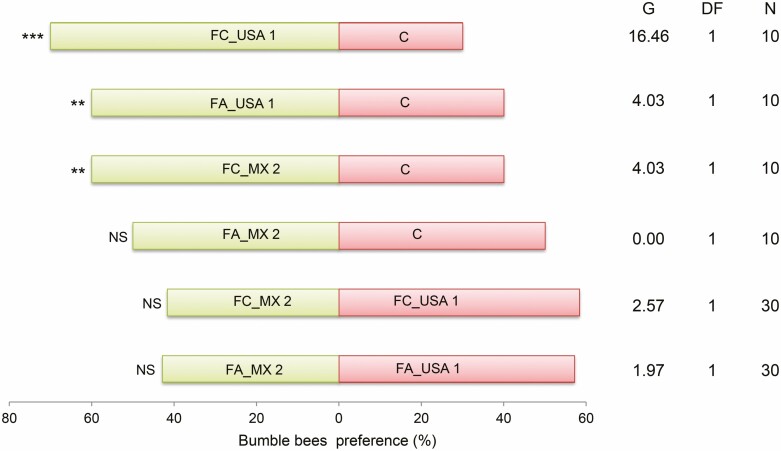
Preference of *Bombus impatiens* for floral extracts from Mexican and US populations of *Solanum rostratum* in Y-olfactometer (percentage). We performed the following treatments: (1) USA 1 whole flower extract (FC_USA 1) vs control (C), (2) USA 1 feeding anther extract (FA_USA 1) vs control (C), (3) flower extract MX 2 whole flower (FC_MX 2) vs control (C), (4) MX 2 anther feeding (FA_MX 2) vs control (C), (5) MX 2 whole flower extract (FC_MX 2) vs whole flower extract from USA 1 (FC_USA 1) and (6) FA from MX 2 (FA_MX 2) vs extract from FA from USA 1 (FA_USA 1). G = statistical test, DF = freedom degree, *n* = number of bumble bees per treatment. Significance levels are: ^***^P < 0.001; ^**^P < 0.01; ^*^P < 0.05; ^NS^*P* > 0.05.

Most bumble bees preferred flower extracts rather than the control hexane (*P* < 0.001). Bumble bees showed similar preferences for floral extracts from Mexican and US populations (Fig. 3; *P* > 0.05).

## Discussion

Volatiles present in floral scents are essential signals in the plant–pollinator interactions, mediating pollinators’ choices (Soler *et al.* 2011; Suchet *et al.* 2011; Farré-Armengol *et al.* 2015; Larue *et al.* 2016). Our study demonstrates that there is a variation in the volatile components of the floral scents from Mexican and US populations of *S. rostratum.* The Y-olfactometer experiment shows that pollinators preferred the floral extracts over control. This result also agrees with Solís-Montero *et al.* (2018) who demonstrated that olfactory signals influence the preference of *B. impatiens* for FA. The heteranthery in *S. rostratum* involves chemical differentiation in proportion of compounds from the specialized anthers, which facilitates pollinator preference for FA over PA (Solís-Montero *et al.* 2018).

Although we found variation in floral scents between two distribution ranges, all populations were similarly attractive to pollinators in Y-olfactometer experiment. Contrary to the field cage experiment, pollinator preferred to visit plants from US populations rather than those from Mexican populations. The conflicting results can be explained by two possible explanations. One possibility is the effect of the volatile extraction method. Solvent extraction often affects the determination of plant volatile organic compounds (VOCs) due to residual organic solvents (Kong *et al.* 2012*a*; Kong *et al.* 2012*b*). However, this method of extraction is still widely used in industry and research (Hernández-Carmona *et al.* 2013; Chemat and Boutekedjiret 2015; Escorsim *et al.* 2018). One advantage of solvent extraction is that it can extract various compounds with different polarities (low, medium and high) depending on the solvents used (Paibon *et al.* 2011). A previous study by Vega-Polanco *et al.* (2020) on *S. rostratum* florivory employed solid-phase microextraction (SPME) as an extraction technique and found some majority of compounds reported by Solís-Montero *et al.* (2018) that also used hexane extraction. Our results coincide with both studies because we found some of those compounds (see Table 1); however, it is possible that missing compounds in floral extracts influence pollinator preference. Another explanation is that other signals we did not evaluate in this study affect pollinator preference. In a previous study, flower morphology was measured in six Mexican populations of *S. rostratum* (Solís-Montero and Vallejo-Marín 2017). One of these populations, which grew at the same site where we collected seed from MX 2 population, recorded the smallest flower size but more widely sexual organ separation (i.e. herkogamy) than the other five Mexican populations. Flower size could possibly explain why MX 2 population receives few visits (see Fig. 2A), but further studies are needed to confirm this idea. Multimodal signals are learned and memorized better than single ones (Rowe *et al.* 1999). When visual cues are offered in conjunction with a compound stimulus, the odour is the prominent stimulus that overlaps with the other signals’ memory (Bogdany 1978). Thus, scents can improve attention for other discrimination task, such as colour (Larue *et al.* 2016). Scents can increase colour evaluation by facilitating memory (Burkle and Runyon 2017). Further investigation is necessary to evaluate other signals affecting pollinator preference, such as flower size, colour or shape, and its variation among the range of distribution in *S. rostratum*.

Although floral scents are not the only trait involved in pollinator attraction, these are important in attracting pollinators. For example, pollinators associated more rapidly olfactory signals with floral rewards in comparison to visual cues (Dobson 1994; Wright and Schiestl 2009). For *S. rostratum*, which depends on buzz-pollinating bees for reproduction, being able to attract novel pollinators (i.e. buzz-pollinating bee taxa that have not before encountered *S. rostratum*) could facilitate its establishment outside the plant’s native range. Many invasive plants indeed recruit pollinators in newly occupied habitat establishing novel interactions and successfully integrating into the native pollination networks (Lopezaraiza-Mikel *et al.* 2007; Aizen *et al.* 2008; Morales and Traveset 2008; Vilà *et al.* 2009; Junker *et al.* 2010; Pyšek *et al.* 2011; Lockwood *et al.* 2013), which help plants in the invasion process (Stout and Tiedeken 2017). Invasive populations of *S. rostratum* studied in this work (USA 1 and USA 2) seem to have successfully recruited new pollinators as they maintain similarly high outcrossing rates across invasive ranges in both Mexico (Solís-Montero 2013) and China (Zhang and Lou 2018), as in native Mexican populations (Vallejo-Marín *et al.* 2013).

The variety of volatile compounds that mediate the different biotic interactions is responsible for the functional diversity of scents (Knudsen *et al.* 2006; Muhlemann *et al.* 2014; Junker 2016). We found qualitative differences in floral scents of native and invasive populations of *S. rostratum*. For instance, γ-decalactone is only present in S. *rostratum* from Mexican populations, while *trans*-geranyl acetone is present in *S. rostratum* from US populations. In this study, we observed that the main difference among the floral scent emitted by *S. rostratum* from the native and invasive populations was the relative amount of the main volatiles present in the FA compared to those of the PA, confirming a previous report (Solís-Montero *et al.* 2018). Previous studies have reported differences in the proportions of the compounds that influence the behaviour of arthropods (Dobson 1994; Tan and Nishida 2012; Solís-Montero *et al.* 2018).

The difference in floral volatile composition detected between Mexican and US populations observed here in plants grown in a common garden suggests that volatile variation has a genetic component (de Manincor *et al.* 2022). Little is known about the intraspecific sources of variation in volatiles and how specific environmental conditions affect this variation (Raguso *et al.* 2015). Future studies could compare volatiles emitted in field conditions with those emitted in greenhouse conditions to detect phenotypic plasticity across multiple populations (de Manincor *et al.* 2022).

## Conclusion

Our study found intraspecific variation in the floral scents of plants from the Mexican and US distribution ranges of *S. rostratum*. Although bumble bees visited plants from US populations more frequently than they did with plants from Mexican populations, we found no difference in the preference of *B. impatiens* for floral extracts. Other signals, besides floral volatiles, should be further evaluated to explain the pollinator preference for plants from US populations. Moreover, evaluation of volatiles *in situ* is also recommendable as it may reveal information on this intraspecific variation of floral scent and its interaction with the resident pollinators present in each geographical distribution range.

## Supporting Information

The following additional information is available in the online version of this article –

**Figure S1**. Bioassay of preference of *Bombus impatiens* in a field cage. A: location of *Solanum rostratum* plants within the field cage, B: marking of left and right floral morphs of *S. rostratum* exposed to bumble bees; and C: bumble bee visiting a flower during the bioassay.

**Figure S2**. Bioassay of preference of *Bombus impatiens* for the floral extracts from *Solanum rostratum* in a Y-type olfactometer. A: square wooden cage covered with black cloth and flow meter coupled to the olfactometer for bioassays realization; B: location of the olfactometer inside the wooden box and C: olfactometer using red light ready for the development of bioassays.

**Table S1**. Variable importance from random forest fit of volatile compounds in floral structures of Mexican and the USA populations of *S. rostratum*.

plad049_suppl_Supplementary_Material

## Data Availability

The data used in this manuscript are available and openly downloadable and citable in Mendeley Data at https://dx.doi.org/ doi: 10.17632/gtwj7jn58f.1.

## Notes

**Populations and Communities. Chief Editor:** Jean Burns
