# Supplementary material for: Intraspecific variation of scent and its impact on pollinators’ preferences
Source: AoB Plants. 2023 Jul 21;15(4):plad049. doi: 10.1093/aobpla/plad049 (PMC10407983; doi:10.1093/aobpla/plad049)
Supplement: plad049_suppl_Supplementary_Material [file plad049_suppl_supplementary_material.pdf]

Figure S1

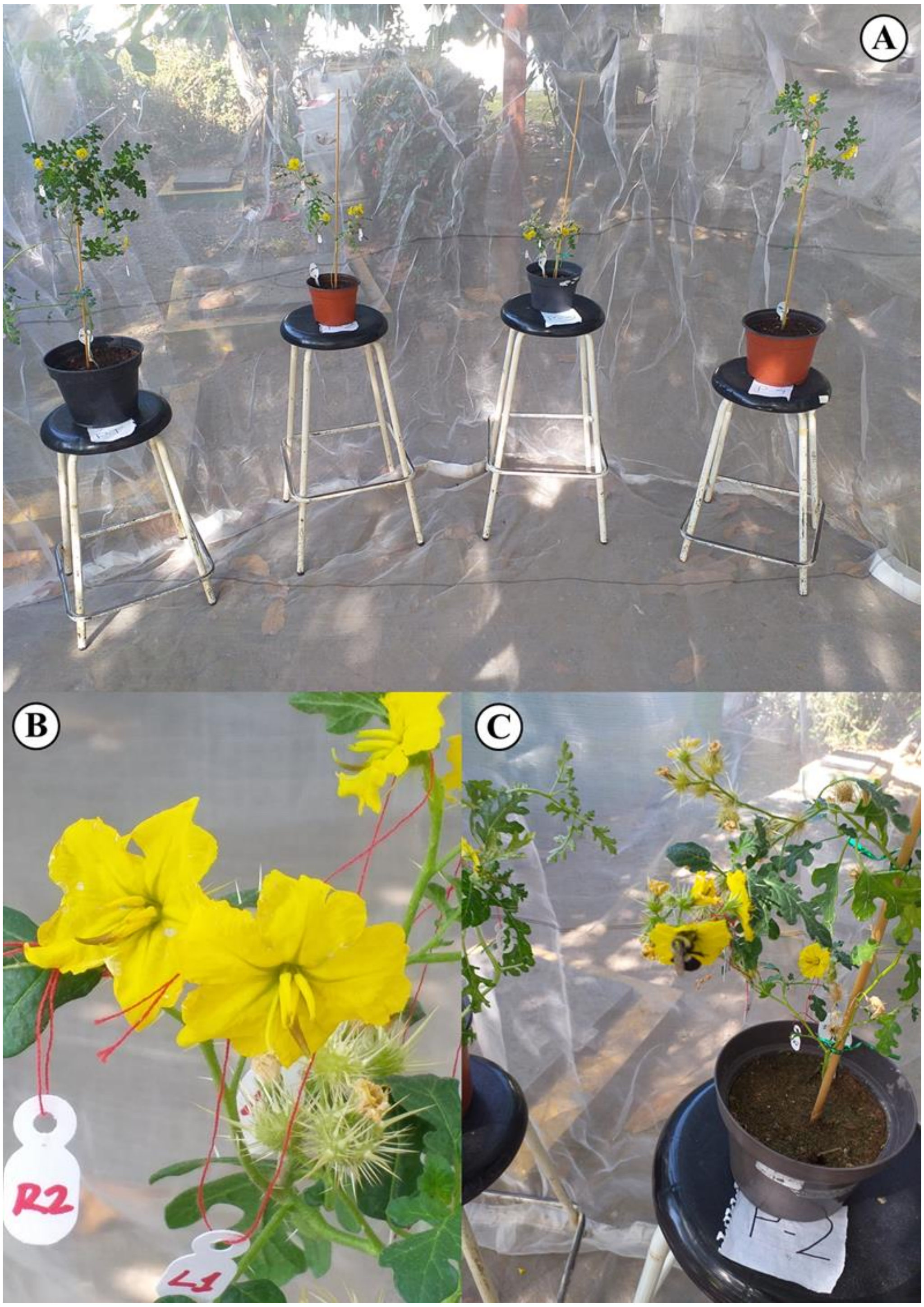

Figure S2

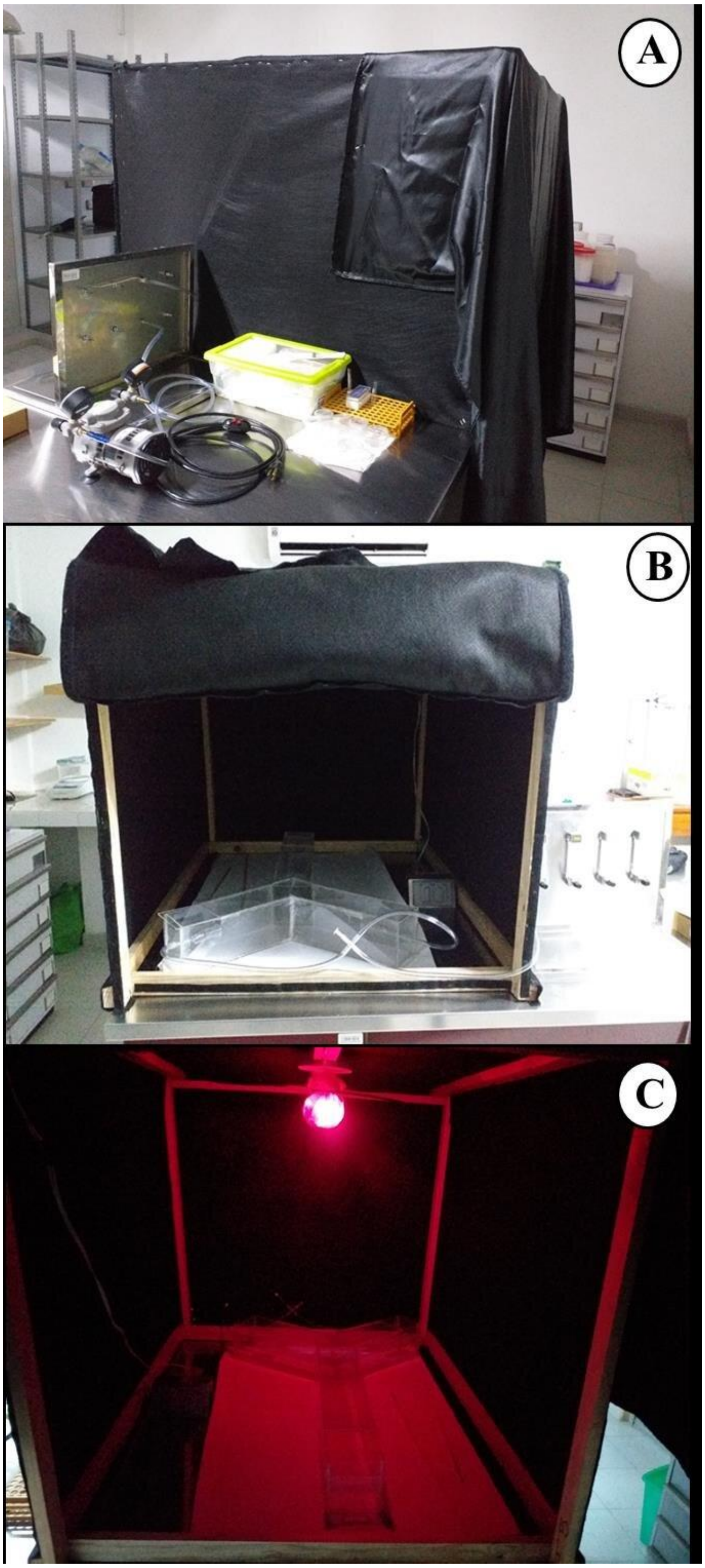

Table S1

ID	Constituents	Gini Index (mean decrease)
**C1**	Dodecane	1.226183
**C2**	Methyl salicylate	0.971983
**C3**	Tetradecane	0.885726
**C4**	Eugenol	1.649198
**C5**	Copaene	0.728848
**C6**	Methyleugenol	0.930051
**C7**	{152 [M]+, 151 (100); 123 (35); 109 (35); 81 (30)}	1.520397
**C8**	trans-Geranylacetone	2.005002
**C9**	γ-Decalactone	1.549263
**C10**	Hexadecane	1.194469
**C11**	(*E*,*E*)-Farnesol	1.530330
**C12**	(*E*,*Z*)-Farnesol	2.099073
**C13**	Pentadecanol	0.894412

Figure S1. Bioassay of preference of *Bombus impatiens* in a field cage. A: location of *Solanum rostratum* plants within the field cage, B: marking of left and right floral morphs of *S. rostratum* exposed to bumble bees; and C: bumble bee visiting a flower during the bioassay.

Figure S2. Bioassay of preference of *Bombus impatiens* for the floral extracts from *Solanum rostratum* in a Y-type olfactometer. A: square wooden cage covered with black cloth and flow meter coupled to the olfactometer for bioassays realization; B: location of the olfactometer inside the wooden box and C: olfactometer using red light ready for the development of bioassays.

Table S1. Variable importance from random forest fit of volatile compounds in floral structures of Mexican and the USA populations of *S. rostratum*.
